# *Msx* Homeobox Genes Critically Regulate Embryo Implantation by Controlling Paracrine Signaling between Uterine Stroma and Epithelium

**DOI:** 10.1371/journal.pgen.1002500

**Published:** 2012-02-23

**Authors:** Shanmugasundaram Nallasamy, Quanxi Li, Milan K. Bagchi, Indrani C. Bagchi

**Affiliations:** 1 Department of Comparative Biosciences, University of Illinois Urbana-Champaign, Urbana, Illinois, United States of America; 2 Department of Molecular and Integrative Physiology, University of Illinois Urbana-Champaign, Urbana, Illinois, United States of America; Baylor College of Medicine, United States of America

## Abstract

The mammalian *Msx* homeobox genes, *Msx1* and *Msx2*, encode transcription factors that control organogenesis and tissue interactions during embryonic development. We observed overlapping expression of these factors in uterine epithelial and stromal compartments of pregnant mice prior to embryo implantation. Conditional ablation of both *Msx1* and *Msx2* in the uterus resulted in female infertility due to a failure in implantation. In these mutant mice (*Msx1/2* ^d/d^), the uterine epithelium exhibited persistent proliferative activity and failed to attach to the embryos. Gene expression profiling of uterine epithelium and stroma of *Msx1/2* ^d/d^ mice revealed an elevated expression of several members of the *Wnt* gene family in the preimplantation uterus. Increased canonical Wnt signaling in the stromal cells activated β-catenin, stimulating the production of a subset of fibroblast growth factors (FGFs) in these cells. The secreted FGFs acted in a paracrine manner via the FGF receptors in the epithelium to promote epithelial proliferation, thereby preventing differentiation of this tissue and creating a non-receptive uterus refractory to implantation. Collectively, these findings delineate a unique signaling network, involving Msx1/2, Wnts, and FGFs, which operate in the uterus at the time of implantation to control the mesenchymal-epithelial dialogue critical for successful establishment of pregnancy.

## Author Summary

During implantation, various tissue compartments within the uterus, including epithelium and stroma, undergo sequential proliferation and differentiation as the embryo attaches to the uterus and invades into the maternal tissue. There is only limited understanding of the molecular signaling pathways that interconnect these tissue compartments to achieve a functional state of the uterus conducive to implantation. This study reveals that a unique signaling network regulated by the homeobox transcription factors MSX1 and MSX2 in the mouse uterus critically controls female fertility. Targeted mutation of *Msx1* and *Msx2* genes in female mice, which results in infertility, established that these factors suppress signaling by the morphogenic ligands, WNTS, in the uterus. In the absence of *Msx1* and *Msx2*, the WNT signaling is elevated, leading to the production of a subset of fibroblast growth factors (FGFs) in uterine stroma. These FGFs act in a paracrine manner on the uterine epithelium to promote epithelial proliferation, which results in lack of uterine receptivity and implantation failure. This work, therefore, uncovers an important mechanism in mammalian reproduction and development by identifying key paracrine signals that arise from the uterine stroma to control epithelial function during implantation.

## Introduction

Successful implantation is dependent on a timely progression of a series of biological events during which the embryo undergoes functional interactions with the uterus prepared by the maternal factors [1]–[4]. During implantation, various tissue compartments within the uterus, including luminal epithelium, glandular epithelium, and stroma, undergo sequential proliferation and differentiation as the embryo attaches to the luminal epithelium and invades into the stroma. In mice, the luminal and glandular epithelial cells are initially in a proliferative state on days 1 and 2 of pregnancy. As pregnancy proceeds, these cells exit from the cell cycle and enter a differentiation program that allows their transition to a receptive state. The stromal cells adjacent to the epithelium begin to proliferate on day 3 and this proliferation becomes widespread following embryo attachment to the receptive luminal epithelium on day 4 of pregnancy [1]–[4]. As the embryos invade through the luminal epithelium into the stromal compartment, the stromal cells differentiate into secretory decidual cells, which support further growth and development of the implanted embryos until placentation ensues [1]–[4].

Extensive research over the past decade, using genetically altered mutant mouse models, has identified several factors that critically regulate uterine function in the preimplantation or postimplantation phases of pregnancy [5]–[11]. However, there is only limited insight into the molecular mechanisms and signaling pathways that interconnect the various cellular compartments of the uterus to achieve receptivity to embryo implantation. Recent studies in our laboratory indicated that a subset of fibroblast growth factors (FGFs) produced by the stromal cells act in a paracrine manner to promote luminal epithelial proliferation. The transcription factor Hand2 suppresses the production of these FGFs and inhibits luminal epithelial proliferation at the time of implantation [11]. Studies by Lee *et al* identified Indian hedgehog (IHH) as an epithelial paracrine factor that acts on the stromal cells to regulate their differentiation [7]. These studies support the concept that maternal competency for implantation is determined by a critical exchange of diffusible signals between the epithelial and stromal compartments, allowing transition of these tissues to proper functional states that permit embryo attachment and invasion. Identification of epithelial or stromal transcription factors and their downstream molecular pathways that control these signals is essential for a clear understanding of the molecular basis of implantation.

It was previously reported that the messenger RNA encoding the homeobox transcription factor MSX1 is expressed in the peri-implantation uterus [12]. We observed that MSX2, another member of MSX family, is expressed in a similar pattern in the epithelial and stromal compartments of the preimplantation uterus during days 1–4 of pregnancy. Expressions of MSX1 and MSX2 were markedly reduced in both compartments following embryo attachment. These findings raised the possibility that the pathways regulated by MSX1 or MSX2 or both regulate the receptive state of the preimplantation uterus. Global deletion of *Msx1* and *Msx2* gene is embryonic lethal, necessitating the development of conditional deletion of these genes to study their functions during implantation. Conditional ablation of either *Msx1* or *Msx2* showed only modest impairment in embryo implantation, resulting in sub-fertility of the mutant mice. On the other hand, conditional ablation of both *Msx1* and *Msx2* in mouse uterus led to complete infertility due to a failure of embryo attachment to the uterine epithelium. We further established that *Msx*1 and *Msx*2 function by suppressing the expression of several members of the *Wnt* family. In *Msx1/Msx2*-null uterus, continued expression of a subset of WNTs enhances β-catenin signaling in the stroma, which in turn induces the expression of specific members of the fibroblast growth factor (FGF) family in this compartment. One or more of these FGFs act via the FGF receptors in the glandular and luminal epithelial tissues to promote proliferation and prevent differentiation. Lack of differentiation of the glandular epithelial cells results in the failure to express critical factors, such as the leukemia inhibitory factor (LIF), which are critical for implantation. Additionally, undifferentiated luminal epithelial cells exhibit persistent expression of MUC-1, a glycoprotein that serves as a maternal barrier to the attachment of the embryo. This study, therefore, delineated a novel signaling network downstream of *Msx*1 and *Msx*2, mediating the stromal-epithelial crosstalk critical for successful establishment of pregnancy.

## Results

### *Msx*1 and *Msx*2 are expressed in the preimplantation uterus

**Figure 1:**
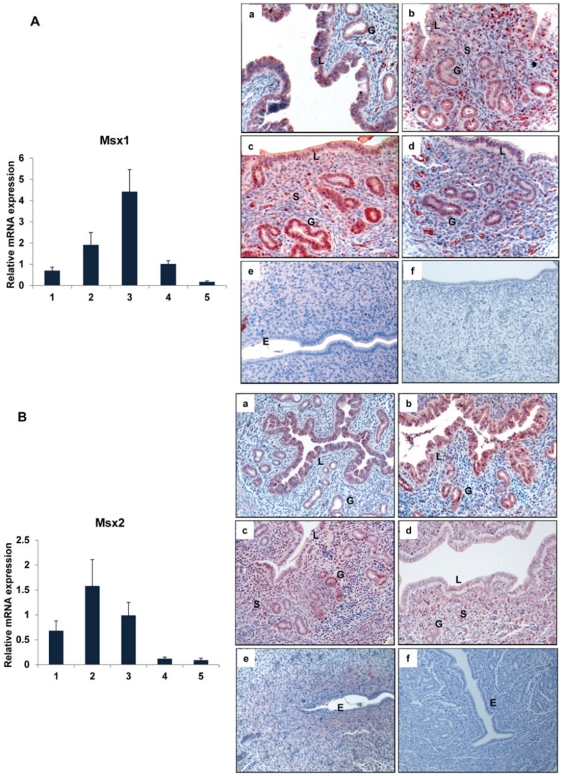
Expression of *Msx*1 and *Msx*2 in the uterus during early pregnancy. Real-time PCR was performed to monitor the expression of mRNAs corresponding to *Msx1* and *Msx2* in uterus on days 1 to 5 of gestation. The relative levels of gene expression on different days of pregnancy were determined by setting the expression level of *Msx1* mRNA (A, Left panel) and *Msx2* mRNA (B, Left panel) on day 1 of pregnancy at 1.0. *Rplp0*, encoding a ribosomal protein, was used to normalize the level of RNA. Uterine sections from day 1 to day 5 (a–e) of pregnancy were subjected to immunohistochemical analysis using anti-MSX1 (A, Right panel) and anti-MSX2 (B, Right panel) antibodies. Panel f shows uterine sections from day 3 pregnant mice treated with non-immune IgG. L, G and S indicate luminal epithelium, glandular epithelium and stroma, respectively.

The spatio-temporal profiles of mRNAs and proteins corresponding to *Msx1* and *Msx2* were examined in the mouse uterus during the preimplantation phase by real-time PCR and immunohistochemistry (IHC), respectively. The expression of both *Msx*1 and *Msx*2 mRNAs followed a similar pattern: an increase on days 2–3 of pregnancy followed by a decline on day 4 at the time of embryo implantation (Figure 1A, left panel; Figure 1B, left panel). Both MSX1 and MSX2 proteins were expressed in uterine epithelium on day 1 of pregnancy (Figure 1A, panel a; Figure 1B, panel a). The expression of these proteins increased on days 2 and 3 of pregnancy and was localized to both glandular epithelium and stroma (Figure 1A, panels b and c; Figure 1B, panels b and c). The expression of MSX1 and MSX2 proteins then declined on day 4 at the time of embryo implantation and were undetectable on day 5 (Figure 1A, panels d & e; Figure 1B, panels d & e). Therefore, similar expression of *Msx*1 and *Msx*2 was observed in the uterine epithelial and stromal compartments in the preimplantation phase.

### Ablation of *Msx1* and *Msx2* in the uterus leads to infertility

To investigate the function of *Msx*1 and *Msx*2 in the uterus, we employed the Cre-LoxP strategy to create conditional single knockout of *Msx1* or *Msx2* or double knockout of *Msx1* and *Msx2* in the uteri of adult mice. Transgenic mice expressing Cre under the control of progesterone receptor (PR) promoter was previously used to ablate “floxed” genes selectively in cells expressing PR, including uterine cells [7]–[11]. We, therefore, crossed the PR-Cre mice with mice harboring the “floxed” *Msx1* or *Msx2* or both to create *Msx1^d/d^*, *Msx2^d/d^* or *Msx1^d/d^Msx2^d/d^* mice. We confirmed the deletion of *Msx1* or *Msx2* in the uteri of these mutant mice by real-time PCR and IHC. As shown in Figure S1, neither *Msx1/Msx2* mRNA nor MSX*1*/MSX2 protein was detected in uteri of *Msx1^d/d^Msx2^d/d^* mice on day 3 of pregnancy, confirming successful abrogation of both *Msx* genes in uteri of *Msx1^d/d^Msx2^d/d^* mice.

A six-month breeding study demonstrated that the single mutant females, *Msx1^d/d^* and *Msx2^d/d^*, are subfertile but the double mutant females, *Msx1^d/d^Msx2^d/d^*, are completely infertile (Table 1). The subfertility of the single mutant *Msx1^d/d^* or *Msx2^d/d^* was likely due to compensation of the function of one *Msx* gene by the other. Indeed, in *Msx1*-null uteri, the level of *Msx2* expression in the uterus was markedly elevated (Figure S2).

**Table 1:** Ablation of uterine *Msx*1 and *Msx*2 leads to female infertility.

Genotype	No. of animals	No. of Litters born	No. of litters per animal(Mean ± SEM)	No. of pups born	No. of pups per litter(Mean ± SEM)
Msx1^f/f^Msx2^f/f^	6	32	5.3±0.2	261	8.1±0.4
Msx1^d/d^	6	14	2.8±0.8	64	4.5±0.6
Msx2^d/d^	6	22	3.6±0.6	132	6.0±0.5
Msx1^d/d^Msx2^d/d^	6	0	0	0	0

The results of a six-month breeding study are shown.

While *Msx1^f/f^Msx2^f/f^* mice exhibited normal litter size and pregnancy rates, the *Msx1^d/d^Msx2^d/d^* females failed to become pregnant when mated with wild-type males. However, copulatory plugs were observed upon mating, indicating normal mating behavior. To investigate the cause of infertility in *Msx1^d/d^Msx2^d/d^* females, we examined their ovarian functions by inducing superovulation. Prepubertal *Msx1^f/f^Msx2^f/f^* and *Msx1^d/d^Msx2^d/d^* mice were treated with a regimen of gonadotropin hormones as described in Materials and Methods. We observed that, upon gonadotropin stimulation, the number of eggs produced by *Msx1^d/d^Msx2^d/d^* was comparable to that produced by the *Msx1^f/f^Msx2^f/f^* females (Figure S3A), indicating that ovulation is not affected in the absence of *Msx1* and *Msx2*. To further examine the ovulation and fertilization in these mice under normal physiological conditions, blastocysts were recovered from uteri of *Msx1^f/f^Msx2^f/f^* and *Msx1^d/d^Msx2^d/d^* mice on day 4 of pregnancy prior to implantation. Once again, no significant difference was found in either the number or the morphology of the embryos recovered from *Msx1^f/f^Msx2^f/f^* and *Msx1^d/d^Msx2^d/d^* uteri (Figure S3B and Figure 3C). In further support of normal ovarian activity, the serum levels of progesterone and estrogen were comparable in *Msx1^f/f^Msx2^f/f^* and *Msx1^d/d^Msx2^d/d^* females on day 4 of pregnancy (Figure S3D and Figure S3E). Collectively, these results suggested that the infertility of *Msx1^d/d^Msx2^d/d^* females is not due to impairment in the hypothalamic-pituitary-ovarian axis or lack of fertilization, but is likely due to defective implantation or pregnancy failure following implantation.

### Ablation of *Msx1* and *Msx2* in the uterus affects embryo attachment to the luminal epithelium

**Figure 2:**
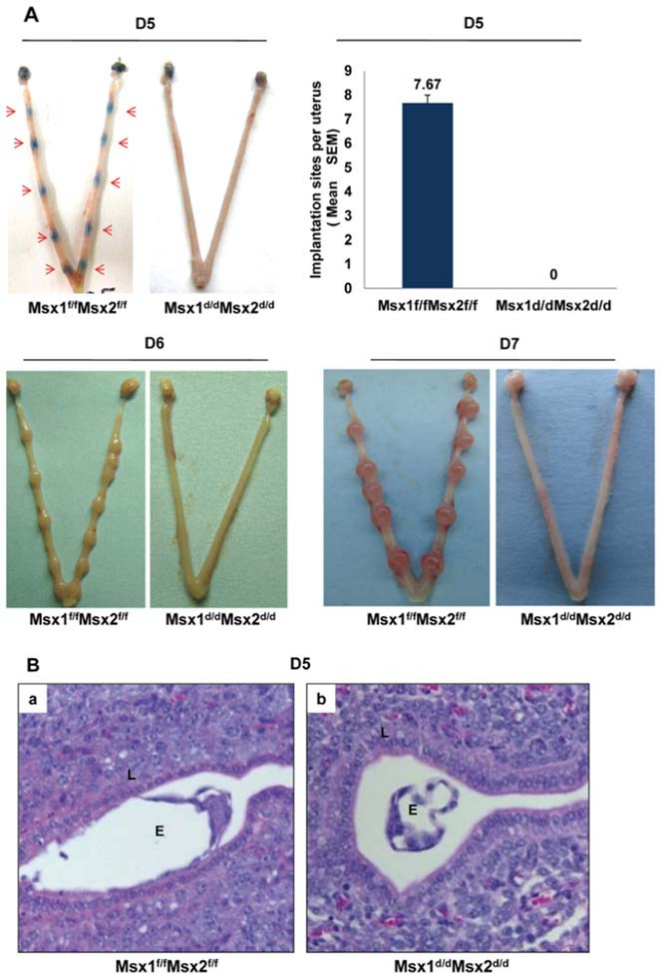
Lack of uterine *Msx*1 and *Msx*2 causes implantation failure. A. Embryo implantation sites were examined in *Msx1^f/f^Msx2^f/f^* and *Msx1^d/d^Msx2^d/d^* mice by the vascular permeability assay, which can be scored as distinct blue bands (red arrows) following an injection of Chicago blue dye on day 5 of pregnancy (D5, n = 6) or direct eye-visualization of implanted embryo on day 6 (D6, n = 4) and on day 7 (D7, n = 4) of pregnancy. The graph represents the quantification of implantation sites in *Msx1^f/f^Msx2^f/f^* and *Msx1^d/d^Msx2^d/d^* mice on day 5 of pregnancy. B. Failure of embryo attachment in *Msx1^d/d^Msx2^d/d^* uteri. Histological analysis of uterine sections obtained from *Msx1^f/f^Msx2^f/f^* (a) and *Msx1^d/d^Msx2^d/d^* (b) mice on day 5 (n = 3) of pregnancy by Hematoxylin and Eosin staining. Note the intimate contact between embryo and luminal epithelium in *Msx*1*^f/f^Msx*2*^f/f^* mice and the free floating embryo in the uterine lumen of *Msx*1*^d/d^Msx*2*^d/d^* mice. L and E indicate luminal epithelium and embryo respectively.

In mice, the attachment of the embryos to the uterine wall initiates the process of implantation. This is accompanied by increased vascular permeability at the implantation sites, which can be scored visually as distinct blue bands following an intravenous injection of Chicago blue dye [13]. As shown in Figure 2A, *Msx1^f/f^Msx2^f/f^* mice displayed distinct implantation sites on day 5 of pregnancy. In contrast, the *Msx1^d/d^Msx2^d/d^* females did not show any sign of implantation. Implanted embryos were also assessed on days 6 and 7 of pregnancy by visual inspection. Our results indicated that implantation sites are absent in *Msx1^d/d^Msx2^d/d^* uteri (Figure 2A).

Histological analysis of *Msx1^f/f^Msx2^f/f^* females on day 5 of pregnancy showed, as expected, a close contact of embryonic trophectoderm with uterine luminal epithelium (Figure 2B, panel a). In contrast, in *Msx1^d/d^Msx2^d/d^* uteri, embryos did not attach to luminal epithelium. Instead, blastocysts remained free-floating in the lumen and were readily recovered by uterine flushing of the *Msx1^d/d^Msx2^d/d^* females (Figure 2B, panel b). Taken together, these results indicated that the loss of *Msx1* and *Msx2* expression in the uterus resulted in the inability of the luminal epithelium to acquire competency for embryo implantation.

### Estrogen receptor activity is elevated in uterine epithelium of *Msx1^d/d^Msx2^d/d^* mice at the time of implantation

**Figure 3:**
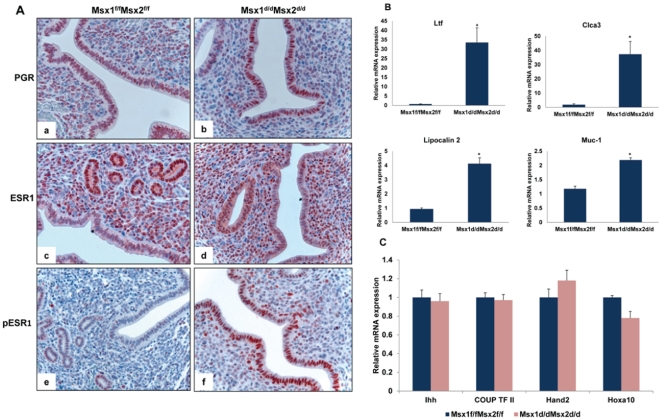
Enhanced ESR1 activity in the luminal epithelium of *Msx1^d/d^Msx2^d/d^* uteri. A. Uterine sections obtained from *Msx*1*^f/f^ Msx*2*^f/f^* (left panel) and *Msx*1*^d/d^Msx*2*^d/d^* (right panel) mice on day 4 of pregnancy were subjected to IHC using antibodies against PGR (top panel, a and b), ESR1 (middle panel, c and d) and phospho-ESR1 (lower panel, e and f). B. Real-time PCR was performed to analyze the expression of E-regulated genes, lactotransferrin (*Ltf*), Clca3, lipocalin2 and Muc-1 in uteri of *Msx*1*^f/f^Msx*2*^f/f^* and *Msx*1*^d/d^Msx*2*^d/d^* mice on day 4 of pregnancy. The level of *Ck18* was used as internal control to normalize gene expression. The data are represented as the mean fold induction ± SEM, *p<0.05. C. Real-time PCR was performed to analyze the expression of P-regulated genes, Ihh, COUP-TF II, Hand2 and Hoxa10, in uteri of *Msx*1*^f/f^Msx*2*^f/f^* and *Msx1^d/d^Msx2^d/d^* mice on day 4 of pregnancy. The level of *Rplp0* or *Ck18* was used as internal control to normalize gene expression.

In mice, the window of uterine receptivity is critically regulated by the steroid hormones 17β-estradiol (E) and progesterone (P), acting through their cognate nuclear receptors. We, therefore, examined the expression levels of progesterone receptor (PGR), estrogen receptor alpha (ESR1), and their downstream genes in the uteri of *Msx1^d/d^Msx2^d/d^* mice by immunohistochemistry and real-time PCR analyses. As shown in Figure 3A, the expression levels of PGR (top panel) and ESR1 (middle panel) proteins in the luminal epithelium or stromal compartment of *Msx1^d/d^Msx2^d/d^* uteri were comparable to those of *Msx1^f/f^Msx2^f/f^* controls. However, we noted that the expression of the transcriptionally active form of ESR1, phosphorylated at serine 118 [14], was markedly up-regulated in the luminal epithelial cells of *Msx1^d/d^Msx2^d/d^* uteri, indicating that ER activity is elevated in the uterine epithelia of these mice (lower panel). This observation indicated that the pathways directed by *Msx1/Msx2* play an important role in controlling the ESR1 activity, which is normally suppressed in uterine epithelium during the receptive phase [15]–[17]. Consistent with this up-regulation of transcriptional activity of ESR1, expression of mRNAs corresponding to well-known E-regulated genes, such as lactotransferrin (*Ltf*) [18], chloride channel, calcium activated, family member 3 (*Clca3*) [19], lipocalin 2 [20] and mucin 1 (*Muc-1*) [21], was significantly elevated in uterine epithelium of *Msx1^d/d^Msx2^d/d^* uteri on day 4 of pregnancy (Figure 3B).

In contrast, the expression of *Ihh*, a P-responsive gene in uterine epithelium [7] remained unaltered in *Msx1^d/d^Msx2^d/d^* uteri. Additionally, the mRNA levels of *Hand2* [11] and *Hoxa10* [3], well-known P-regulated genes in uterine stroma, and that of chicken ovalbumin upstream promoter-transcription factor II (*COUP-TF II*), a downstream target of IHH in the uterine stroma [9], were unaffected in the uteri of *Msx1^d/d^Msx2^d/d^* mice (Figure 3C). These results indicated that the loss of *Msx1* and *Msx2* did not impact on the transcriptional activity of PGR, but resulted in an enhancement of the epithelial ESR1 function.

**Figure 4:**
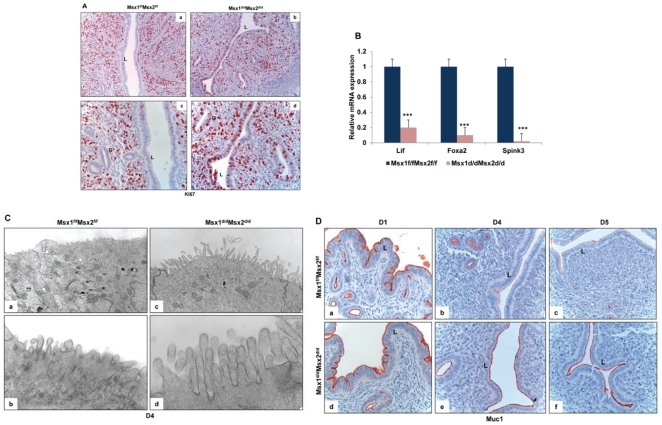
Enhanced proliferation in the uterine epithelium and lack of receptivity in *Msx1^d/d^Msx2^d/d^* mice. A. Immunohsitochemical localization of Ki67 in the uterine sections of *Msx*1*^f/f^Msx*2*^f/f^* (left panel, a and c) and *Msx*1*^d/d^Msx*2*^d/d^* (right panel, b and d) mice on day 4 of pregnancy. Panels a and b indicate lower magnification (20×) and c and d indicate higher magnification (40×). L and G indicate luminal epithelium and glandular epithelium respectively. B. Real-time PCR was performed to analyze the expression of glandular factors, *Lif*, *Foxa2* and *Spink3* in uteri of *Msx*1*^f/f^Msx*2*^f/f^* and *Msx*1*^d/d^Msx*2*^d/d^* mice on day 4 of pregnancy. The level of *Ck18* was used as internal control to normalize gene expression. The data are represented as the mean fold induction ± SEM, ***p<0.0001. C. Transmission electron microscopy of uterine sections obtained from *Msx1^f/f^ Msx2^f/f^* (left panel, a and b) and *Msx1^d/d^Msx2^d/d^* (right panel, c and d) mice on day 4 of pregnancy. Panels a and c indicate lower magnification (5Kx) and b and d indicate higher magnification (30Kx). D. Immunohistochemical analysis of Muc-1 expression in the uterine sections of *Msx*1*^f/f^Msx*2*^f/f^* (upper panel) and *Msx*1*^d/d^Msx*2*^d/d^* (lower panel) mice on day 1 (a and d), day 4 (b and e) and day 5 (c and f) of pregnancy. L indicates luminal epithelium.

A hallmark of the receptive state of normal pregnant uterus is the cessation of epithelial cell proliferation prior to implantation [1]–[4], [22]. Therefore, in *Msx1^f/f^Msx2^f/f^* mice, immunostaining of Ki67, a cell proliferation marker, was undetectable in the uterine luminal and glandular epithelium on day 4 of pregnancy (Figure 4A, panels a and c). However, uterine sections of *Msx1^d/d^Msx2^d/d^* mice exhibited robust immunostaining for Ki67 in the luminal and glandular epithelia (Figure 4A, panels b and d), indicating persistent epithelial cell proliferation on day 4 in the absence of *Msx1* and *Msx2*. Previous studies indicated that the ability of the glandular epithelium to undergo differentiation and produce factors, including leukemia inhibitory factor (LIF) [23], Foxa2 [24], and Spink3 [25], is critical for implantation. As shown in Figure 4B, the expression of these factors was drastically reduced in uteri deficient of *Msx*1 and *Msx*2. Collectively, these findings indicated that persistent proliferation of luminal and glandular epithelia results in impaired epithelial transition from a proliferative to a non-proliferative state that allows proper differentiation. This impairment is a major contributor to implantation failure in *Msx1^d/d^Msx2^d/d^* mice.

Another important parameter of receptive uterus is the membrane transformation of uterine epithelium at the time of implantation. The presence of long microvilli, containing a thick layer of glycoprotein known as the glycocalyx, on the uterine epithelium is indicative of the nonreceptive stage. A marked flattening of these microvilli occurs in the receptive phase prior to implantation [26]. Transmission electron microscopy (TEM) revealed that, in contrast to the control epithelium, the epithelia of *Msx1^d/d^Msx2^d/d^* uteri fail to undergo appropriate remodeling to promote microvilli flattening, indicating impaired uterine receptivity in these mice (Figure 4C).

The impaired functional state of uterine epithelium in *Msx1^d/d^Msx2^d/d^* mice was further confirmed when we analyzed the expression of MUC-1 protein, a major component of the endometrial glycocalyx, during early pregnancy. The expression status of MUC-1 is considered an important indicator of uterine receptivity [21]. As the luminal epithelium differentiates and the uterus achieves receptivity, MUC-1 expression is down regulated in this tissue. Persistent MUC-1 expression is indicative of a non-receptive uterus, which is not conducive to embryo implantation. As shown in Figure 4D, prominent expression of MUC-1 was detected in the uterine epithelia of control *Msx1^f/f^Msx2^f/f^* mice on day 1 of pregnancy (panel a). As the pregnancy advanced to days 4 (panel b) and 5 (panel c), Muc-1 was progressively down regulated in uterine epithelia of these mice, consistent with the attainment of receptive status. In contrast, an intense expression of MUC-1 was observed in uteri of *Msx1^d/d^Msx2^d/d^* mice on days 4 and 5 (panels d–f). Therefore, elevated epithelial ESR1 signaling, which likely triggered persistent expression of MUC-1 in luminal epithelium, disrupted uterine receptivity, resulting in implantation failure in *Msx1^d/d^Msx2^d/d^* mice.

### *Msx1/Msx2* regulates WNT and FGF signaling in the uterus

**Figure 5:**
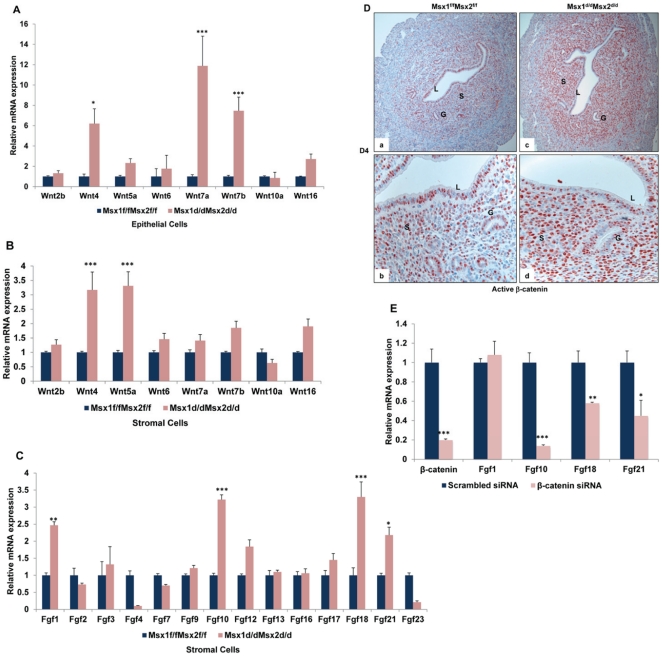
Wnt/β-catenin signaling controls FGF synthesis in uterine stromal cells. A. Real-time PCR was performed to analyze the expression of Wnt ligands in uterine epithelial cells of *Msx1^f/f^Msx2^f/f^* and *Msx1^d/d^Msx2^d/d^* mice on day 4 of pregnancy. The level of *Ck18* was used as internal control to normalize gene expression. The data are represented as the mean fold induction ± SEM, *p<0.01, ***p<0.0001. B. Real-time PCR was performed to analyze the expression of Wnt ligands in uterine stromal cells of *Msx1^f/f^Msx2^f/f^* and *Msx1^d/d^Msx2^d/d^* mice on day 4 of pregnancy. C. Real-time PCR was performed to analyze the expression of Fgf family members in uterine stromal cells of *Msx1^f/f^Msx2^f/f^* and *Msx1^d/d^Msx2^d/d^* mice on day 4 of pregnancy. The level of *Rplp0* was used as internal control to normalize gene expression. The data are represented as the mean fold induction ± SEM, *p<0.01, **p<0.001, ***p<0.0001. D. The level of active β-catenin in uterine sections of *Msx1^f/f^Msx2^f/f^* (left panel) and *Msx1^d/d^Msx2^d/d^* (right panel) mice on day 4 of pregnancy was analyzed by IHC. (Magnification: a and c: 10×, b and d: 40×) E. Primary stromal cells were isolated from uteri of *Msx1^d/d^Msx2^d/d^* mice on day 3 of pregnancy and transfected with siRNA targeted to the β-catenin mRNA. Total RNA was isolated 24 h after transfection to analyze the expression of Fgf family members by Real-time PCR. The level of *Rplp0* was used as an internal control to normalize gene expression. The data are represented as the mean fold induction ± SEM, *p<0.01, **p<0.001, ***p<0.0001.

To gain insights into the mechanisms underlying the implantation defect of uteri lacking *Msx1* and *Msx2*, we isolated luminal epithelial and stromal cells from *Msx1^f/f^Msx2^f/f^* and *Msx1^d/d^Msx2^d/d^* uteri on day 4 of pregnancy and performed compartment-specific gene expression profiling, using Affymetrix Mouse GeneChip arrays. Interestingly, our study revealed up-regulation of two distinct classes of signaling factors, WNTs and FGFs, in *Msx1^d/d^Msx2^d/d^* uteri compared to *Msx1^f/f^Msx2^f/f^* uteri. The microarray data (GEO accession #GSE30969) were validated by real-time PCR analysis. In the epithelial compartment of *Msx1^d/d^Msx2^d/d^* uteri, we observed stimulated expression of mRNAs corresponding to several Wnts, including *Wnt4*, *Wnt7a* and *Wnt7b* (Figure 5A). In the stromal cells of *Msx1^d/d^Msx2^d/d^* uteri, we observed marked up-regulation of *Wnt4* and *Wnt5a* mRNAs (Figure 5B). In addition, the levels of mRNAs encoding several members of the FGF family, such as *Fgf1*, *Fgf10*, *Fgf18* and *Fgf21*, were elevated in uterine stromal cells as a consequence of deletions of *Msx1* and *Msx2* (Figure 5C). The expression of mRNAs corresponding to several other FGF family members as well as other growth factors, such as HBEGF, EGF, IGF-1, and, HGF which are expressed in the uterus during pregnancy, was not significantly altered in the uterine stroma of *Msx1^d/d^Msx2^d/d^* mice (Figure 5C and Figure S4).

We next investigated whether the increased expression of the Wnt ligands in the uteri of *Msx1^d/d^Msx2^d/d^* mice is translated into increased activation of the Wnt signaling pathway. Wnt signals are transduced via the canonical Wnt/β-catenin-dependent pathway or the non-canonical β-catenin-independent pathways [27]–[29]. When we examined the expression of active β-catenin in uterine sections of *Msx1^f/f^Msx2^f/f^* and *Msx1^d/d^Msx2^d/d^* mice on day 4 of pregnancy, we noted comparable levels of nuclear expression of active β-catenin in luminal and glandular epithelium in both genotypes (Figure 5D). However, a marked increase in the level of nuclear β-catenin was observed in the stromal cells of *Msx1Msx2*-null uteri, indicating that canonical β-catenin signaling is markedly enhanced in the *Msx1Msx2*-ablated stroma.

Interestingly, previous studies indicated that the production of FGFs, particularly FGF10 and FGF18, is stimulated downstream of canonical Wnt signaling during certain cellular processes, such as chick embryo development, bone development and human hepatocellular carcinoma [30]–[32], raising the possibility that the enhanced β-catenin signaling seen in uterine stromal cells of *Msx1^d/d^Msx2^d/d^* mice may drive the increased FGF synthesis in these cells. To test this possibility, primary stromal cells were isolated from uteri of *Msx1^d/d^Msx2^d/d^* mice on day 3 of pregnancy and transfected with siRNA targeted specifically to the β-catenin mRNA. We observed that treatment with this siRNA resulted in more than 80% reduction in β-catenin mRNA expression compared to cells transfected with control (scrambled) siRNA (Figure 5E). Most importantly, as shown in Figure 5E, siRNA-mediated down regulation of β-catenin in the stromal cells led to a significant reduction in expression of FGF10, FGF18, and FGF21. However, the expression of FGF1 remained unaltered in cells treated with β-catenin siRNA. These results indicated that canonical Wnt signaling via β-catenin regulates the expression of a specific subset of FGF family members in the uterine stromal cells.

**Figure 6:**
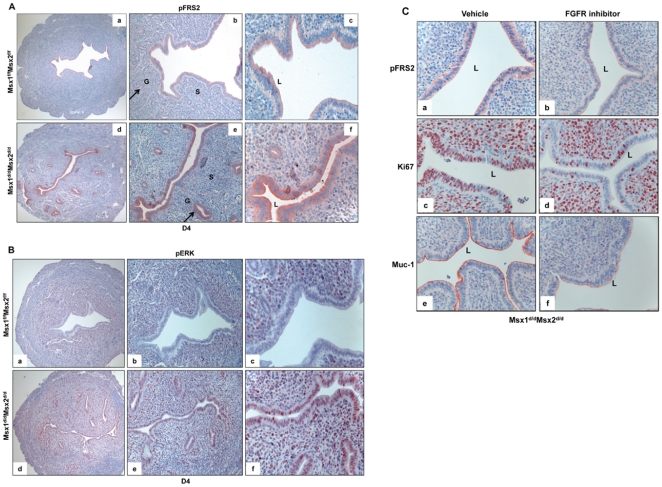
Enhanced FGFR signaling in the epithelium of *Msx1^d/d^Msx2^d/d^* uteri. A. The level of p-FRS2 was examined in the uterine sections of *Msx1^f/f^Msx2^f/f^* (upper panel) and *Msx1^d/d^Msx2^d/d^* (lower panel) mice on day 4 of pregnancy by immunohistochemistry. Magnification: a and d: 10×, b and e: 20×, c and f: 40×. B. The level of p-ERK was examined in the uterine sections of *Msx1^f/f^Msx2^f/f^* (upper panel) and *Msx1^d/d^Msx2^d/d^* (lower panel) mice on day 4 of pregnancy by immunohistochemistry. Magnification: a and d: 10×, b and e: 20×, c and f: 40×. L, G and S indicate luminal epithelium, glandular epithelium, and stroma respectively. C. FGFR-specific inhibitor PD173074 was applied to one uterine horn of *Msx1^d/d^Msx2^d/d^* (n = 3) mice on day 3 of pregnancy. The other horn served as vehicle-treated control. Uterine horns were collected on day 4 morning and sections were subjected to immunohistochemistry to detect p-FRS2, Ki67, and Muc-1.

We next investigated whether the increased production of FGFs downstream of Wnt signaling leads to enhanced FGF receptor (FGFR) signaling in the uteri of *Msx1^d/d^Msx2^d/d^* mice. Stimulation of the cell surface FGFRs by FGF ligands leads to phosphorylation of specific tyrosine residues in a critical docking protein, FGFR substrate 2 (FRS2), which guides the assembly of distinct multi-protein complexes, leading to the activation of either MAP kinase or AKT signaling cascades [33]–[35]. We, therefore, investigated the state of activation of the FGFR signaling pathway in the uteri of *Msx1^f/f^Msx2^f/f^* and *Msx1^d/d^Msx2^d/d^* mice by monitoring the level of phospho-FRS2. We observed only low level of phospho-FRS2 in the uterine luminal or glandular epithelium or stroma of *Msx1^f/f^Msx2^f/f^* mice on day 4 of pregnancy (Figure 6A, panels a–c). In contrast, a marked elevation in the level of phospho-FRS2 was observed specifically in the luminal and glandular epithelium, but not in the stroma (Figure 6A, panels d–f) of *Msx1Msx2*-null uteri, indicating that FGFR signaling is increased in uterine epithelium in the absence of *Msx*1/*Msx2*. Since the FGFs are produced in the stroma of these mutant uteri, this finding suggests that they act in a paracrine fashion via the FGFRs on the epithelial cells.

The kinases ERK1/2 and/or PI3K/AKT are known to be activated downstream of FGF receptor signaling [33]. We, therefore, investigated whether these pathways were activated in the epithelia of *Msx1Msx2*-ablated uteri. As shown in Figure 6B, phospho-ERK1/2 (pERK) was undetectable in the uterine epithelium of *Msx1^f/f^Msx2^f/f^* mice on day 4 of pregnancy (panels a–c). However, a dramatic increase in the immunostaining of pERK1/2 was seen in epithelium of *Msx1Msx2*-null uteri on day 4 of pregnancy (panels d–f). In contrast, the expression of phospho-AKT was undetectable in both of these genotypes (data not shown), suggesting that the ERK1/2 pathway, rather than the PI3K/AKT pathway, is the key downstream mediator of enhanced FGFR signaling in *Msx1Msx2*-null uteri.

To examine whether the elevated mitogenic activity in the luminal epithelium of *Msx1^d/d^Msx2^d/d^* uteri on day 4 of pregnancy is indeed a result of the enhanced FGF signaling, we administered PD173074, a FGFR-specific inhibitor [36], or vehicle into uterine horns of *Msx1^d/d^Msx2^d/d^* mice in the pre-implantation phase. As shown in Figure 6C, the epithelia of vehicle-treated uterine horns of these mice showed strong expression of phospho-FRS2 on day 4 of pregnancy (panel a). Treatment with the FGFR inhibitor led to a marked reduction in the level of phospho-FRS2 in the uterine epithelium (Figure 6C, panel b). Concomitant with this down regulation of FGFR signaling, we observed a decline in the proliferative activity of *Msx1Msx2*-null uterine epithelia as well as down-regulation of MUC-1 expression (Figure 6C, panels c–f). Collectively, these results are consistent with the hypothesis that increased FGF production, downstream of Wnt-β-catenin pathway in *Msx1Msx2*-null uterine stroma, stimulates epithelial proliferation by activating FGFR-ERK1/2 signaling pathway. The proliferative epithelium fails to undergo differentiation, resulting in persistent expression of MUC-1, which acts as a major barrier to embryo attachment and implantation.

## Discussion

Members of *Msx* family of homeobox genes, comprising of *Msx*1, *Msx*2, and *Msx*3, are critical regulators of tissue morphogenesis [37]–[39]. While *Msx*1 and *Msx*2 are expressed in several tissues during embryonic development, *Msx*3 expression is mostly restricted to neural tube [40], [41]. The present study describes the expression of *Msx*1 and *Msx2* in adult uterus and addresses their roles in female reproduction. Using mutant mouse models harboring conditional deletion of *Msx1* and/or *Msx2* in the uterus, we established that these factors play critical roles in regulating uterine function during implantation. Due to the overlapping spatio-temporal expression of these two transcription factors in uterine epithelium and stroma, it is not surprising that female mice carrying deletion of either *Msx1* or *Msx2* are subfertile, while those lacking both *Msx1* and *Msx2* are infertile. We observed that the expression of *Msx2* is markedly elevated in *Msx1*-null uterus during early pregnancy (Figure S2), supporting the concept that the loss of function of one *Msx* gene during early pregnancy is partially compensated by the other.

*Msx1* and *Msx2* are critical regulators of the receptive state of the uterus during implantation. Particularly interesting is the finding that uterine expression of *Msx1/Msx2* influences the activity of β-catenin in the stroma, which in turn regulates epithelial activity during early pregnancy. The identity of the factors that function downstream of *Msx1/Msx2* to regulate stromal-epithelial cross-talk during implantation was revealed by compartment-specific gene expression profiling of epithelial and stromal cells collected from control and *Msx*1*Msx2*-ablated uteri. We found that, in the absence of *Msx1* and *Msx2* in the uterus, the expression of several WNT ligands was up-regulated in uterine epithelial and stromal cells. While the expression of WNT4, WNT7a, and WNT7b was elevated in the epithelium, that of WNT4 and WNT5a increased in the stroma. With the exception of WNT5a, these WNTs are known to signal via the canonical pathway to release β-catenin from a complex with GSK3β, leading to its stabilization and nuclear accumulation [27]–[29]. Nuclear β-catenin then associates with TCF/LEF family transcription factors to regulate cellular gene expression. Consistent with this scenario, a marked increase in the level of active β-catenin was observed in uterine stromal cells of *Msx1^d/d^Msx2^d/d^* uteri, while the active β-catenin levels remained unaltered in the surface epithelium. Our results indicated that canonical Wnt signaling is specifically enhanced in the stromal cells as a consequence of *Msx1Msx2* ablation. How does *Msx1/Msx2* regulate the WNTs and whether the β-catenin activation in the stromal cells is driven by WNTs originating in the epithelium or stroma, is unclear.

An important finding of this paper is that, in addition to WNTs, the expression of several members of the FGF family is stimulated in the stromal cells of *Msx1^d/d^Msx2^d/d^* uteri. The FGFs belong to a large family of growth factors, comprising 23 distinct members [33]–[35]. We observed that a subset of FGFs, including FGF1, FGF10, FGF18 and FGF21, exhibited marked up-regulation in uterine stroma of *Msx1^d/d^Msx2^d/d^* mice, indicating that the expression of these growth factors are normally suppressed by *Msx*1/*Msx2*. Interestingly, previous studies have shown that TCF/LEF, activated downstream of WNT-β-catenin signaling in colorectal cancer cells, binds to the promoter regions of FGF18 and FGF20 [30], [31], [42]. Studies have also shown that, in the chick embryo, WNT-β-catenin signaling triggered the synthesis of FGF8 and FGF10, which control the initiation of limb development. These previous findings suggested that WNT-activated β-catenin regulates the expression of a subset of FGFs [30]. In the present study, we provide direct evidence that active β-catenin regulates the synthesis of the FGFs, particularly FGF10, FGF18, and FGF21, in the stromal cells, uncovering a link between the WNT and FGF signaling pathways in the endometrium. The precise mechanism by which active β-catenin regulates the expression of these FGFs in uterine stromal cells remains to be determined.

The FGFs exert their paracrine responses by binding to FGFRs on the surface of the target cells and activating the receptor tyrosine kinase pathway. It is well documented that signaling via FGFRs leads to tyrosine phosphorylation of the docking protein FRS2, followed by the recruitment of multiple distinct complexes, which results in activation of Ras/ERK/MAP kinase and/or PI3 kinase/AKT signaling pathways in a variety of cell types [33], [35]. In uteri lacking *Msx*1 and *Msx2*, the accumulation in the uterine epithelium of phospho-FRS2, a key indicator of FGF signaling, indicated activation of FGFR signaling. Bazer and his coworkers have previously reported that the FGFRs are activated in ovine uterine epithelia of sheep in response to the secretion of FGF7 and FGF10 from the progesterone-primed mesenchyme and proposed that these factors are potential regulators of the maternal-fetal interactions [43], [44]. However, in the mouse uterus, the expression of FGF10, FGF18, and FGF21 is suppressed during the receptive phase of implantation. The expression of these factors is induced in the absence of *Msx*1 and *Msx*2, and the consequent increase in FGFR signaling is associated with the lack of uterine receptivity and implantation failure in *Msx1^d/d^Msx2^d/d^* mice.

The central hypothesis of this paper is that *Msx1/Msx2* controls uterine receptivity at the time of embryo implantation by regulating epithelial function. During normal pregnancy in mice, the uterus attains receptive status on day 4 of gestation when the luminal and glandular epithelia cease to proliferate and begin to differentiate. Our study suggests that, in the absence of *Msx*1 and *Msx2*, the uterine stroma produces a subset of FGFs, which act via the FGFRs to stimulate the ERK1/2 kinase pathway in both luminal and glandular epithelia. As a consequence, the uterine epithelia of *Msx1^d/d^Msx2^d/d^* mice remain proliferative and fail to undergo transformation to the receptive state that allows embryo attachment to initiate implantation. The activation of the ERK1/2 pathway in the epithelium also triggers phosphorylation of epithelial ESR1 at serine-118. It is well established that this phosphorylation event is critical for the transcriptional activation of ESR1 [14]. An elevated ESR1 signaling in the epithelium is, however, detrimental to the implantation process. For example, ESR1 promotes the expression of Muc-1, a well-known cell surface glycoprotein, which creates a barrier that prevents embryo attachment. In mice, high levels of MUC-1 are present in the non-receptive uterus on days 1 and 2 of pregnancy. As the pregnancy progresses, MUC-1 expression declines in the epithelium and it is drastically reduced on day 4 at the time of implantation [21]. Therefore, the reduction of MUC-1 expression is considered a sign of uterine receptivity in mice. The persistence of high levels of MUC-1 in the *Msx1^d/d^Msx2^d/d^* uteri on day 4 of pregnancy is indicative of hyperestrogenic activity in the luminal epithelium and, consequently, reflects a lack of uterine receptivity.

Pathways downstream of *Msx1* and *Msx2* also control the synthesis of glandular factors critical for uterine receptivity at the time of implantation. While the uterine luminal epithelium is the initial site of embryo attachment, the glandular epithelium is an important source of paracrine factors required for the establishment and maintenance of pregnancy [45]. As uterus acquires competency for implantation, the glandular epithelial cells cease to proliferate and undergo differentiation to express factors, such as LIF and FOXA2, which are critical for embryo implantation [23], [24]. Presumably due to enhanced WNT and FGF signaling in *Msx1^d/d^Msx2^d/d^* uteri, the glandular epithelial cells remain proliferative and fail to express LIF and FOXA2. Consistent with this hypothesis, a recent study has shown that expression of constitutively active β-catenin in mouse endometrium leads to enhanced proliferation and glandular hyperplasia [46].

We recently reported that the transcription factor HAND2 suppresses the production of a subset of FGFs, which act in a paracrine manner to stimulate the proliferation of the luminal epithelium [11]. Conditional deletion of *Hand2* in the uterus also results in the failure of implantation due to impaired uterine receptivity caused by increased production of FGFs in the stroma. The uterine phenotype of *Hand2* deletion is remarkably similar to those of *Msx1/Msx2* ablation. We, therefore, examined whether *Hand2* is regulated by *Msx1/Msx2* or vice versa. Surprisingly, our studies showed that *Msx1/Msx2* expression is unaltered in *Hand2*–null uteri (Figure S5). Similarly, the loss of *Msx1* or *Msx2* or both did not affect *Hand2* expression in the uterus during implantation (Figure S6). Furthermore, while *Hand2* coordinately suppresses the expression of FGF1, FGF2, FGF9 and FGF18, *Msx1/Msx2* inhibits the expression of FGF1, FGF10, FGF18, and FGF21. Although these results suggest that one or more of these FGFs act in a paracrine manner through the epithelial FGFRs to promote epithelial cell proliferation, the contribution of each these FGFs is unclear and, therefore, it remains to be determined whether *Hand2* and *Msx1/Msx2* function via similar or distinct mechanisms.

**Figure 7:**
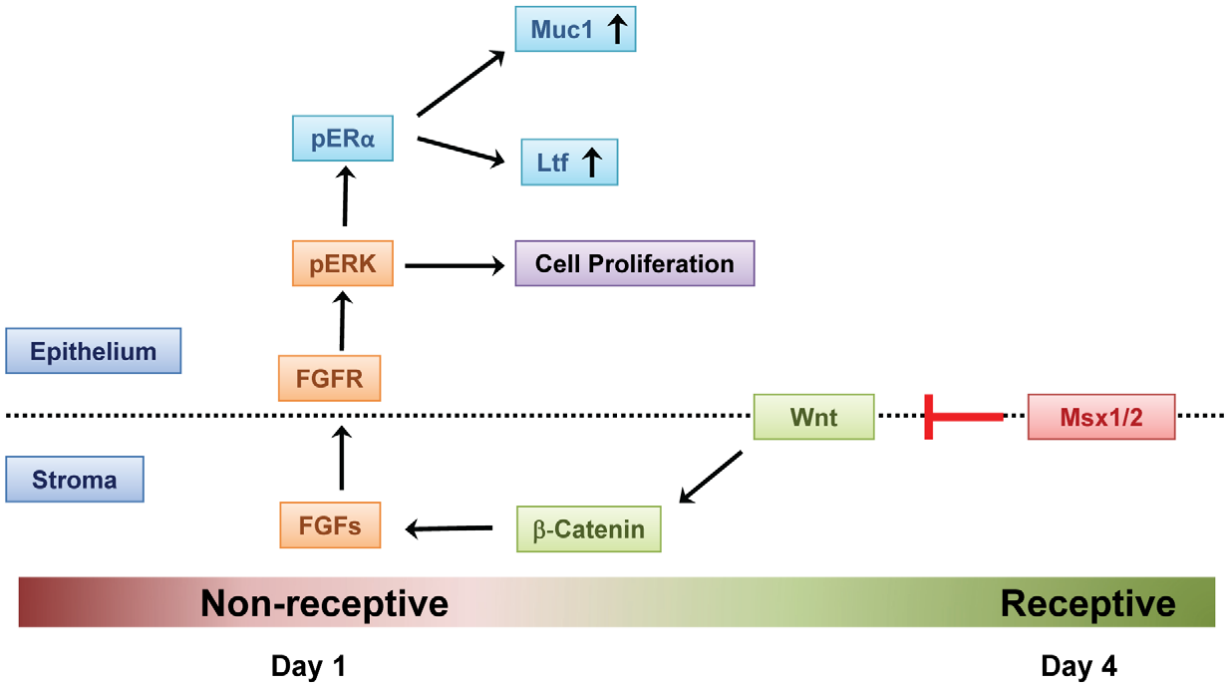
Mechanism of Msx1 and Msx2 action in mouse uterus. In normal pregnancy, MSX1 and MSX2 act to repress WNT and β-catenin signaling and inhibit FGF synthesis in the uterine stroma, thereby suppressing stromal-epithelial cross-talk. In the absence of MSX1 and MSX2, FGFs are induced, activating the epithelial FGFR-ERK1/2 pathway, and promoting epithelial proliferation. Activated ERK1/2 then phosphorylates epithelial ESR1. This triggers transcriptional activation of ESR1 and expression of its target genes, such as *Muc-1*, which prevent the functional transformation of the luminal epithelium to receptive state, blocking embryo implantation.

In summary, we have uncovered a novel mechanism by which *Msx1/Msx2* regulates epithelial function at the time of implantation. In normal pregnancy, these factors act to repress WNT and β-catenin signaling and inhibit FGF synthesis in the uterine stroma, thereby attenuating the paracrine mechanisms that promote epithelial proliferation. It is also evident that the activation of ERK1/2 kinase pathway downstream of FGFR signaling in the epithelium of *Msx1Msx2*-ablated uteri activates transcriptional function of ESR1, contributing to the non-receptive status of the uterus (Figure 7). Continued analysis of the mechanisms by which *Msx*1 and *Msx2* control the WNT-β-catenin-FGF pathway to direct uterine stromal-epithelial communication will clarify our understanding of the molecular events that underlie uterine receptivity.

## Materials and Methods

### Animals

Mice were maintained in the designated animal care facility at the College of Veterinary Medicine of the University of Illinois, Urbana-Champaign, according to the institutional guidelines for the care and use of laboratory animals. To generate the conditional *Msx1Msx2*-null mice (*Msx1^d/d^Msx2^d/d^*), *Msx1Msx2*-floxed (*Msx1^f/f^Msx2^f/f^*) [47] mice were mated with PR-Cre knock-in mice [48].

For breeding studies, cycling *Msx1^d/d^Msx2^d/d^* and *Msx1^f/f^Msx2^f/f^* female mice (C57BL/6 genetic background) were housed with wild-type C57BL/6 male mice (Charles Rivers) for 6 months. The presence of a vaginal plug after mating was designated as day 1 of pregnancy. The number of litters and pups born were recorded at birth to assess the fertility status.

To induce superovulation, 3-week old female mice were administered intraperitoneally with 5 IU of pregnant mare serum gonadotrophin (PMSG, Sigma St. Louis, MO) followed by 5 IU of human chorionic gonadotropin (hCG, Sigma St. Louis, MO) 48 hours later. The mice were killed 16–18 hours post-hCG administration and the oocytes were recovered from the ampulla and counted.

To collect blastocysts, 8-week old female mice were mated with wild-type males. To assess the pre- implantation development of embryos, blastocysts were flushed from day 4 pregnant uteri and examined for their quality under a stereo-zoom microscope.

For certain experiments, the FGFR-specific inhibitor, PD173074 (Selleck Chemicals Co., Ltd., London ON, Canada), was dissolved in DMSO and was diluted with HBSS. Ten microlitre of inhibitor (50 µM) was injected intraluminally in one horn and vehicle was injected in the other horn of *Msx1^d/d^Msx2^d/d^* mice on day 3 of pregnancy. Uterine tissues were collected on day 4 of pregnancy.

### Transmission electron microscopy

Uterine tissues isolated from *Msx*1^f/f^ *Msx*2^f/f^ and *Msx*1*^d/d^Msx*2*^d/d^* female mice on day 4 of pregnancy were fixed in 2.0% paraformaldehyde and 2.5% glutaraldehyde in buffer containing 0.1 M sodium cacodylate. Tissues were then washed and fixed with 1.0% aqueous osmium tetroxide in 0.1 M sodium cacodylate buffer. Following dehydration with ethanol and propylene oxide, the tissues were embedded in 100% Polybed 812 mixture. Sections (80 nm) were cut with an Ultramicrotome, stained and examined under a Philips CM 200 Transmission Electron Microscope.

### Isolation of uterine epithelial and stromal cells

Uterine epithelial cells were isolated as previously described [49]. Briefly, uterine horns were dissected into 3–4 mm pieces and incubated in a solution of 1% trypsin (Difco, Dertroit, MI) in Hank's balanced salt solution (HBSS) for 90 min at 4°C and then for 30 min at room temperature. The tissues were then rinsed with 10% FBS. Under a dissecting microscope, each enzyme treated piece of uterus was squeezed by forceps to separate the epithelium from the rest of the uterine tissue. Uterine stromal cells were isolated as previously described [50]. Briefly, uterine horns of pregnant mice were dissected and placed in HBSS containing 6 g/liter dispase and 25 g/liter pancreatin for 1 h at room temperature and then 15 min at 37°C to remove the endometrial epithelial clumps. The tissues were then placed in HBSS containing 0.5 g/liter collagenase for 45 min at 37°C to disperse the stromal cells. After vortexing, the contents were passed through a 70-µm gauze filter (Millipore). The filtrate contained the stromal cells.

### Culture of uterine stromal cells

The uterine stromal cells, isolated as described above, were diluted in Dulbecco's modified Eagle's Medium-F12 medium (DMEM-F12; with 100 unit/liter penicillin, 0.1 g/liter streptomycin, 1.25 mg/liter Fungizone) with 2% heat-inactivated fetal calf serum. The live cells were counted by trypan blue staining using a hemocytometer. Cells were then seeded in 6-well cell culture plates. The unattached cells were removed by washing several times with HBSS after 2 h, and cell culture was continued after addition of fresh medium supplemented with P (1 µm) and E (10 nm).

### Quantitative real-time PCR analysis (qPCR)

Uterine tissue was homogenized and total RNA was extracted by using TRIZOL reagent, according to the manufacturer's protocol. cDNA was prepared by standard protocols. The cDNA was amplified to quantify gene expression by quantitative PCR, using gene-specific primers and SYBR Green (Applied Biosystems, Warringtom, UK). The expression level of *RPLP0* (*36B4*) or Cytokeratin 18 (*Ck18*) was used as the internal control. For each treatment, the mean Ct and standard deviation were calculated from individual Ct values obtained from three replicates of a sample. The normalized ΔCt in each sample was calculated as mean Ct of target gene subtracted by the mean Ct of internal control gene. ΔΔCt was then calculated as the difference between the ΔCt values of the control and treatment sample. The fold change of gene expression in each sample relative to a control was computed as 2^−ΔΔCt^. The mean fold induction and standard errors were calculated from three or more independent experiments.

### Immunohistochemistry

Uterine tissues were processed and subjected to immunohistochemistry as described previously [13]. Briefly, paraffin-embedded tissues were sectioned at 5 µm and mounted on microscopic slides. Sections were deparaffinized in xylene, rehydrated through a series of ethanol washes, and rinsed in water. Antigen retrieval was performed by immersing the slides in 0.1 M citrate buffer solution, pH 6.0, followed by microwave heating for 25 min. The slides were allowed to cool and endogenous peroxidase activity was blocked by incubating sections in 0.3% hydrogen peroxide in methanol for 15 min at room temperature. After washing with PBS for 15 min and the slides were incubated in a blocking solution for 1 h before incubating them in primary antibody overnight at 4°C with antibodies specific for MSX1 (Abcam, ab73883), MSX2 (Santa Cruz, sc-15396), MUC1 (Novus biological, NB120-15481), Ki67 (BD Pharmingen, 550609), ESR1 (Santa Cruz, sc-7207), p-ESR1 (Santa Cruz, sc-12915), PGR (Neomarkers MS-194-PO), HAND2 (Santa Cruz sc-9409), phospho-FRS2 (R&D systems AF5126) and active β-catenin (PY489, Developmental Studies Hybridoma Bank, Iowa City, IA 52242). The slides were incubated with the biotinylated secondary antibodies at room temperature for 1 h, followed by incubation with horseradish peroxidase-conjugated streptavidin (Invitrogen Corp., MD 21704). The sections were stained in 3-amino-9-ethylcarbazole chromogen (AEC) solution until optimal signal was developed. Sections were counterstained with Mayer's Hematoxylin and examined by bright field microscopy.

### siRNA transfection

Control (scrambled) siRNA and siRNA targeted to β-catenin (s438) were purchased from Ambion Inc. The transfection was performed using SilentFect™ Reagent (Bio-Rad), according to the manufacturer's protocol. The stromal cells were isolated from uteri of *Msx1^d/d^Msx2^d/d^* mice on day 3 of pregnancy and transfected with siRNA after 5–6 h of culture. The cells were harvested 24 h following transfection and RNA was isolated.

### DNA microarray analysis

Uterine epithelial and stromal cells were isolated from *Msx1^f/f^Msx2^f/f^* and *Msx1^d/d^Msx2^d/d^* mice on day 4 of pregnancy. Total RNA was prepared from these cells, and hybridized to Affymetrix GeneChip Mouse Genome 430 2.0 array as previously described [6]. They were processed and analyzed according to the Affymetrix protocol.

### Measurement of serum E and P levels

The levels of E and P in the serum were measured by radioimmunoassay (RIA) performed at the Ligand Core facility of the University of Virginia at Charlottesville.

### Statistical analysis

Statistical analysis was performed by *t*-test or ANOVA. The values were expressed as mean ± SEM and considered significant if *p*<0.05.

## Supporting Information

Figure S1 Loss of *Msx*1 and *Msx*2 expression in the uterus of *Msx*1*^d/d^Msx*2*^d/d^* mice. A. Uterine RNA was extracted from *Msx1^f/f^Msx2^f/f^* and *Msx1^d/d^Msx2^d/d^* mice on day 3 of pregnancy (n = 3) and analyzed by real-time PCR. Relative levels of *Msx*1 and *Msx*2 mRNA expression in uteri of *Msx*1*^d/d^Msx*2*^d/d^* mice are compared to those in *Msx1 ^f/f^Msx2 ^f/f^* control mice. The data are represented as the mean fold induction ± SEM, **p<0.001. B. Uterine sections obtained from day 3 pregnant *Msx*1*^f/f^Msx*2*^f/f^* (left panel) and *Msx*1*^d/d^Msx*2*^d/d^* (right panel) mice were subjected to immunohistochemical analysis. Note the lack of *Msx*1 (upper panel) and *Msx*2 (lower panel) immunostaining in the uteri of the mutant mice. L, G and S indicate luminal epithelium, glandular epithelium and stroma respectively.
(TIF)

Figure S2 Expression of Msx2 is elevated in the uterus of *Msx*1*^d/d^* mice. Left Panel. Uterine RNA was purified from *Msx1^f/f^* and *Msx1^d/d^* mice on day 3 and day 4 of pregnancy and analyzed by real-time PCR. Relative levels of *Msx*2 mRNA expression in uteri of *Msx*1*^d/d^* mice are compared to those in *Msx1^f/f^* control mice. Right Panel. Uterine sections obtained from day 3 and day 4 pregnant *Msx*1*^f/f^* (upper panel) and *Msx*1*^d/d^* (lower panel) mice were subjected to immunohistochemical analysis to detect MSX2. Note the elevated levels of MSX2 immunostaining in the uteri of *Msx*1*^d/d^* mice.
(TIF)

Figure S3 Ovarian functions and preimplantation events remain unaffected in *Msx*1*^d/d^Msx*2*^d/d^* mice. A. Age-matched prepubertal *Msx*1*^f/f^Msx*2*^f/f^* (n = 7) and *Msx*1*^d/d^Msx*2*^d/d^* mice (n = 6) were subjected to superovulation. The oocytes were recovered and counted at 18 h after hCG administration (values are mean ± SEM). B. Pre-implantation embryos were recovered from uteri of *Msx*1*^f/f^Msx*2*^f/f^* (n = 7) and *Msx*1*^d/d^Msx*2*^d/d^* mice (n = 12) in the morning of day 4 of pregnancy, counted (values are mean ± SEM) and photographed. C. Representative morphology of blastocysts recovered from uteri of *Msx*1*^f/f^Msx*2*^f/f^* and *Msx*1*^d/d^Msx*2*^d/d^* mice. D & E: P and E levels in serum of *Msx*1*^f/f^Msx*2*^f/f^* (n = 6) and *Msx*1*^d/d^Msx*2*^d/d^* (n = 10) mice on day 4 of pregnancy. Values are represented as means ± SEM.
(TIF)

Figure S4 Expression of *Egf* family of growth factors in *Msx1^d/d^Msx2^d/d^* uteri. Real-time PCR was performed to monitor the expression of *Egf* family of growth factors in the uterine stroma of *Msx*1*^f/f^ Msx*2*^f/f^* and *Msx*1*^d/d^Msx*2*^d/d^* mice on day 4 of pregnancy.
(TIF)

Figure S5 Msx1 and Msx2 expression in *Hand2^d/d^* uteri. The levels of MSX1 (upper panel) and MSX2 (lower panel) were examined in the uterine sections of Hand*2^f/f^* (left panel) and Hand*2^d/d^* (right panel) mice on day 3 of pregnancy by IHC.
(TIF)

Figure S6 Hand2 expression in *Msx1^d/d^Msx2^d/d^* uteri. The level of Hand2 in uterine sections of *Msx1^f/f^Msx2^f/f^* (left panel) and *Msx1^d/d^Msx2^d/d^* (right panel) mice on day 4 of pregnancy was analyzed by IHC (Magnification: upper panel: 20×, lower panel: 40×).
(TIF)
