# Supplementary figures and images for: Msx Homeobox Genes Critically Regulate Embryo Implantation by Controlling Paracrine Signaling between Uterine Stroma and Epithelium
Source: PLoS Genet. 2012 Feb 23;8(2):e1002500. doi: 10.1371/journal.pgen.1002500 (PMC3285581; doi:10.1371/journal.pgen.1002500)

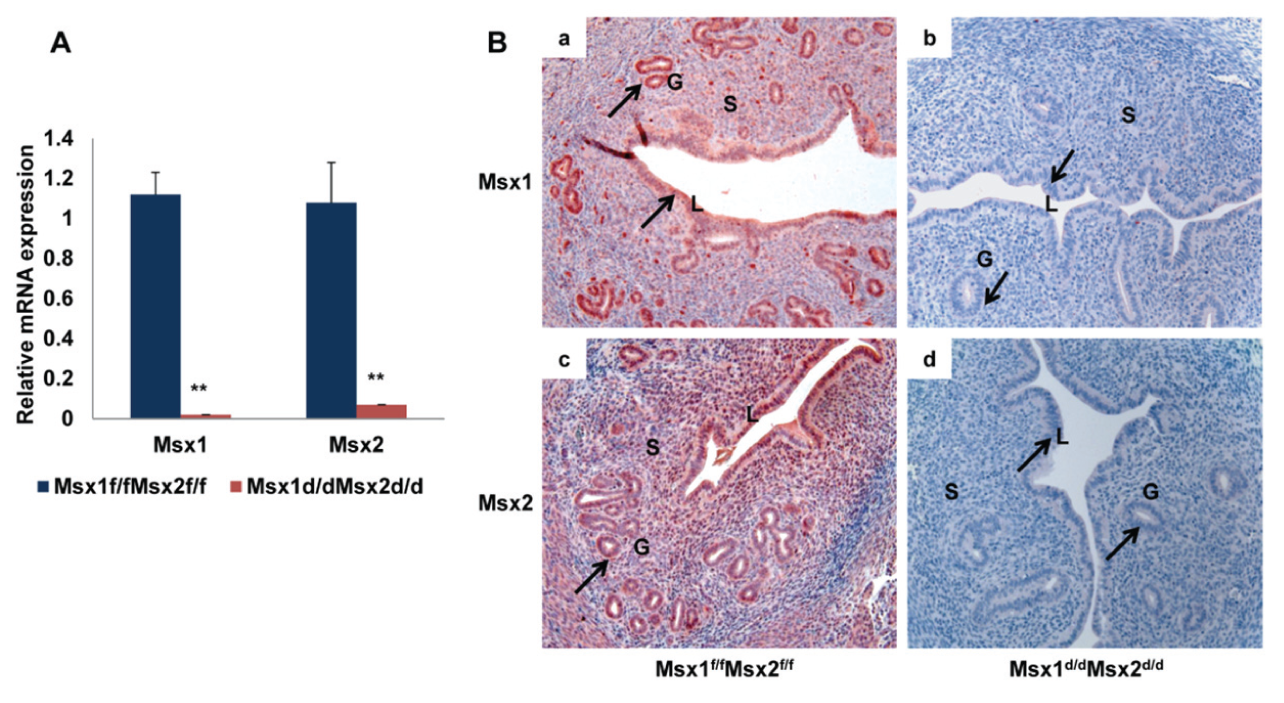

Supplement: Figure S1 — Loss of Msx1 and Msx2 expression in the uterus of Msx1d/dMsx2d/d mice. A. Uterine RNA was extracted from Msx1f/fMsx2f/f and Msx1d/dMsx2d/d mice on day 3 of pregnancy (n = 3) and analyzed by real-time PCR. Relative levels of Msx1 and Msx2 mRNA expression in uteri of Msx1d/dMsx2d/d mice are compared to those in Msx1 f/fMsx2 f/f control mice. The data are represented as the mean fold induction ± SEM, **p<0.001. B. Uterine sections obtained from day 3 pregnant Msx1f/fMsx2f/f (left panel) and Msx1d/dMsx2d/d (right panel) mice were subjected to immunohistochemical analysis. Note the lack of Msx1 (upper panel) and Msx2 (lower panel) immunostaining in the uteri of the mutant mice. L, G and S indicate luminal epithelium, glandular epithelium and stroma respectively. (TIF) [file pgen.1002500.s001.tif]

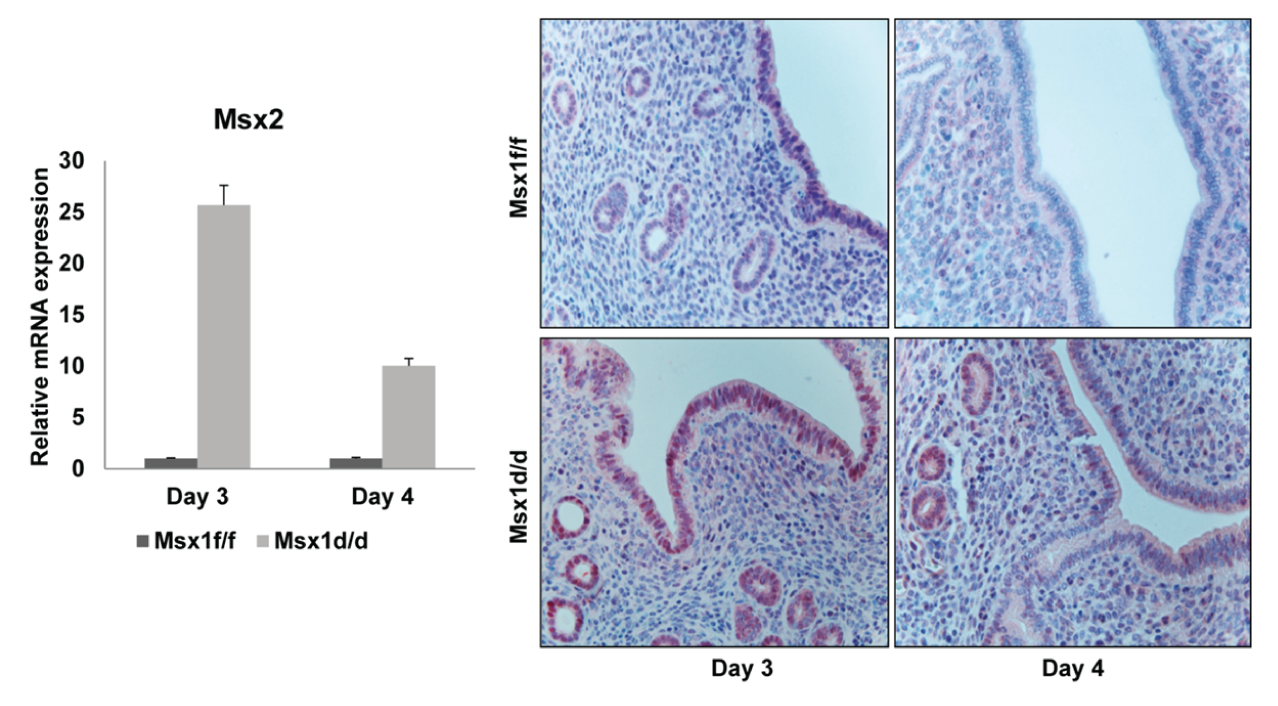

Supplement: Figure S2 — Expression of Msx2 is elevated in the uterus of Msx1d/d mice. Left Panel. Uterine RNA was purified from Msx1f/f and Msx1d/d mice on day 3 and day 4 of pregnancy and analyzed by real-time PCR. Relative levels of Msx2 mRNA expression in uteri of Msx1d/d mice are compared to those in Msx1f/f control mice. Right Panel. Uterine sections obtained from day 3 and day 4 pregnant Msx1f/f (upper panel) and Msx1d/d (lower panel) mice were subjected to immunohistochemical analysis to detect MSX2. Note the elevated levels of MSX2 immunostaining in the uteri of Msx1d/d mice. (TIF) [file pgen.1002500.s002.tif]

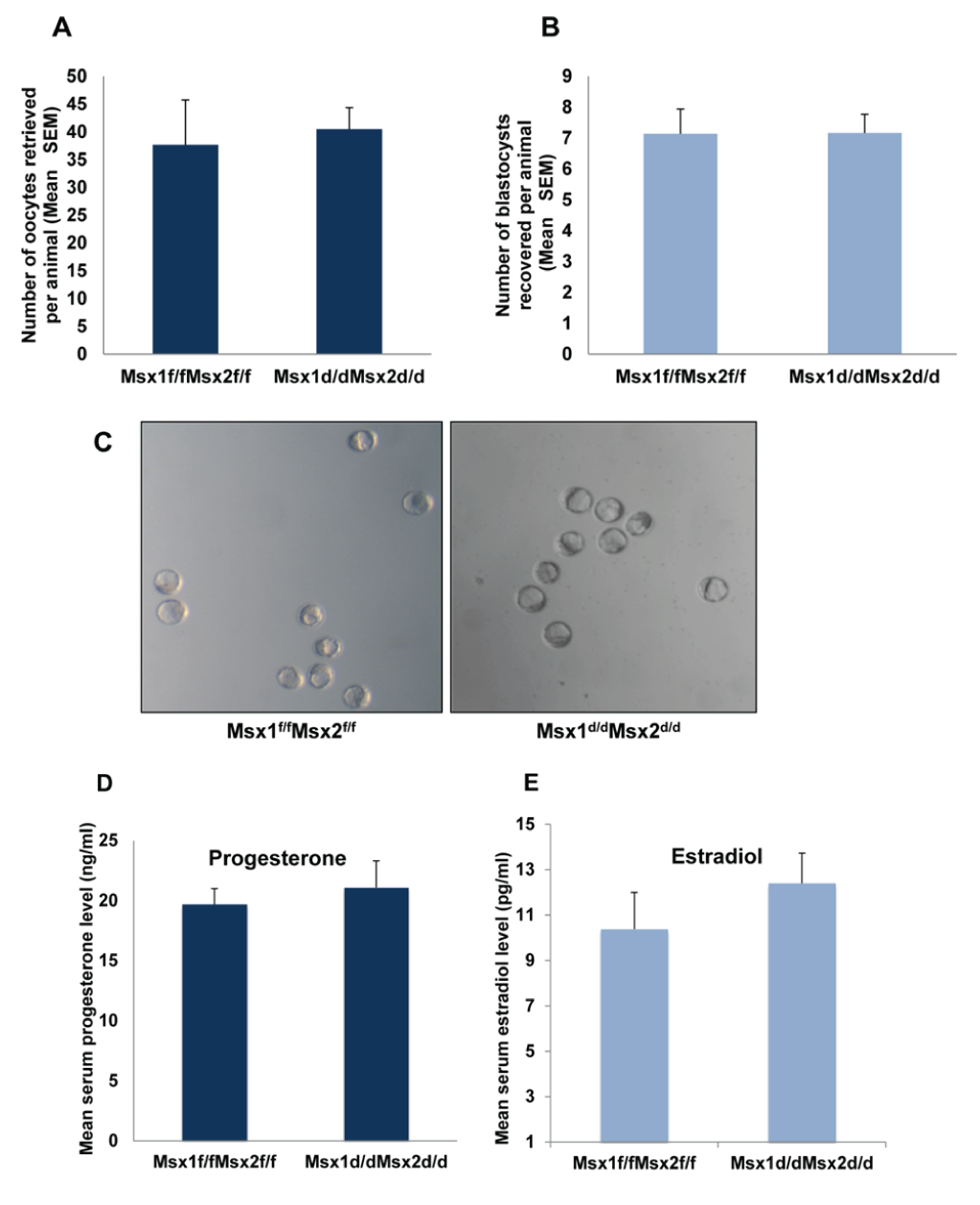

Supplement: Figure S3 — Ovarian functions and preimplantation events remain unaffected in Msx1d/dMsx2d/d mice. A. Age-matched prepubertal Msx1f/fMsx2f/f (n = 7) and Msx1d/dMsx2d/d mice (n = 6) were subjected to superovulation. The oocytes were recovered and counted at 18 h after hCG administration (values are mean ± SEM). B. Pre-implantation embryos were recovered from uteri of Msx1f/fMsx2f/f (n = 7) and Msx1d/dMsx2d/d mice (n = 12) in the morning of day 4 of pregnancy, counted (values are mean ± SEM) and photographed. C. Representative morphology of blastocysts recovered from uteri of Msx1f/fMsx2f/f and Msx1d/dMsx2d/d mice. D & E: P and E levels in serum of Msx1f/fMsx2f/f (n = 6) and Msx1d/dMsx2d/d (n = 10) mice on day 4 of pregnancy. Values are represented as means ± SEM. (TIF) [file pgen.1002500.s003.tif]

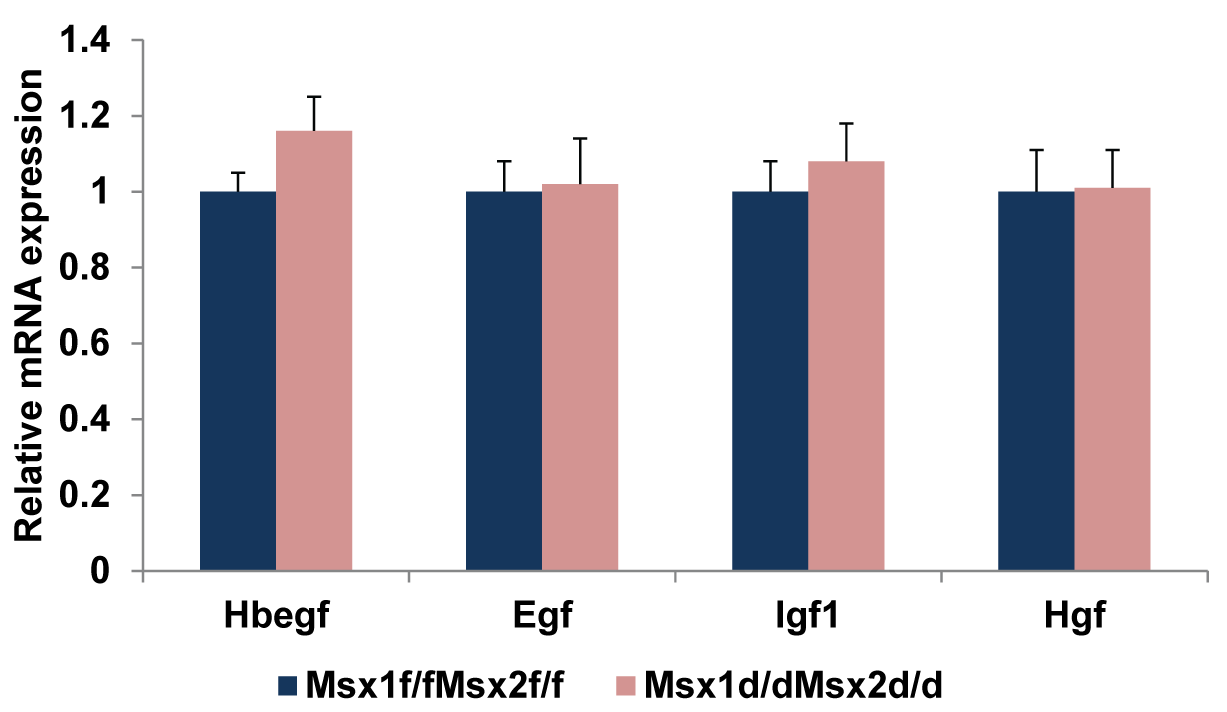

Supplement: Figure S4 — Expression of Egf family of growth factors in Msx1d/dMsx2d/d uteri. Real-time PCR was performed to monitor the expression of Egf family of growth factors in the uterine stroma of Msx1f/f Msx2f/f and Msx1d/dMsx2d/d mice on day 4 of pregnancy. (TIF) [file pgen.1002500.s004.tif]

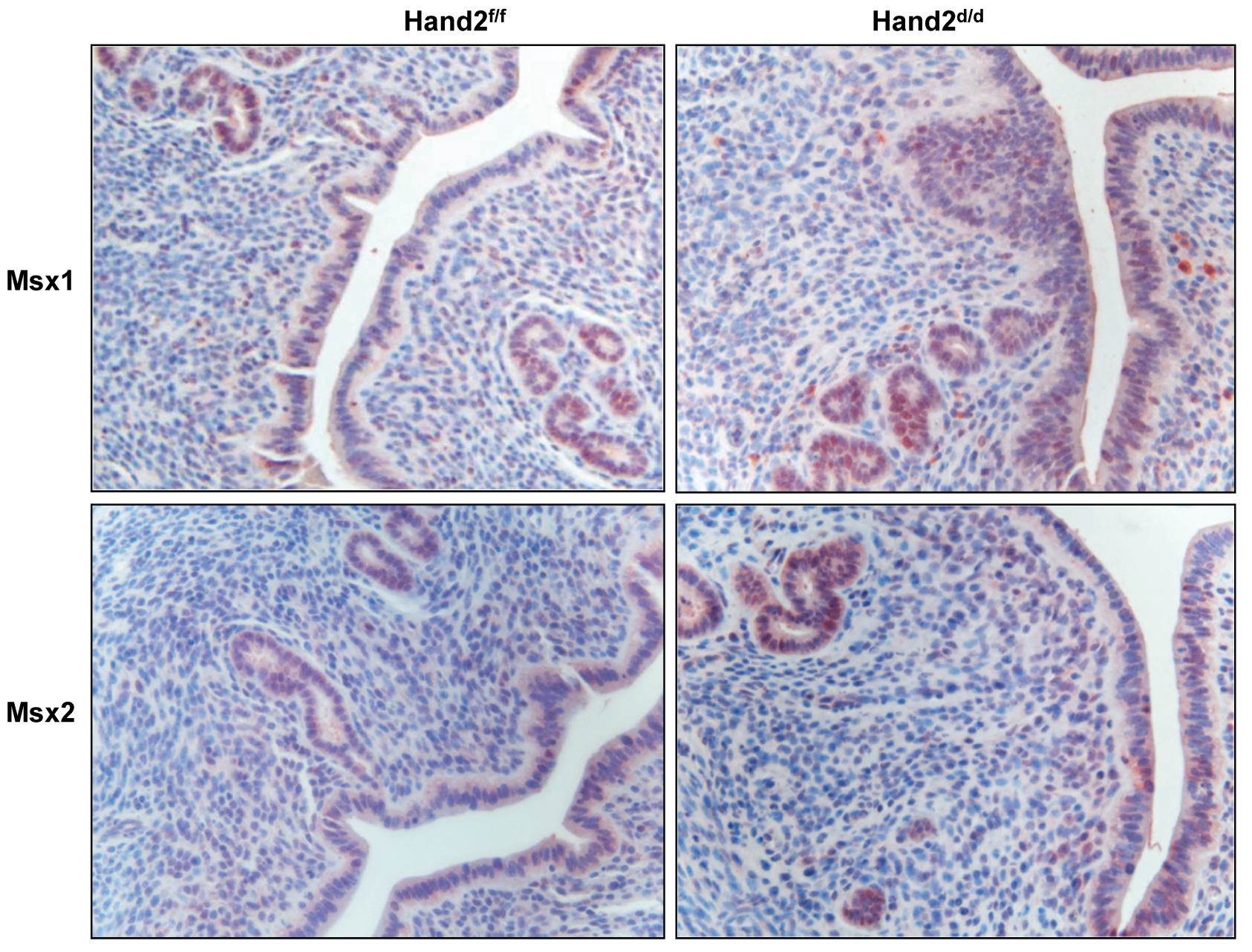

Supplement: Figure S5 — Msx1 and Msx2 expression in Hand2d/d uteri. The levels of MSX1 (upper panel) and MSX2 (lower panel) were examined in the uterine sections of Hand2f/f (left panel) and Hand2d/d (right panel) mice on day 3 of pregnancy by IHC. (TIF) [file pgen.1002500.s005.tif]

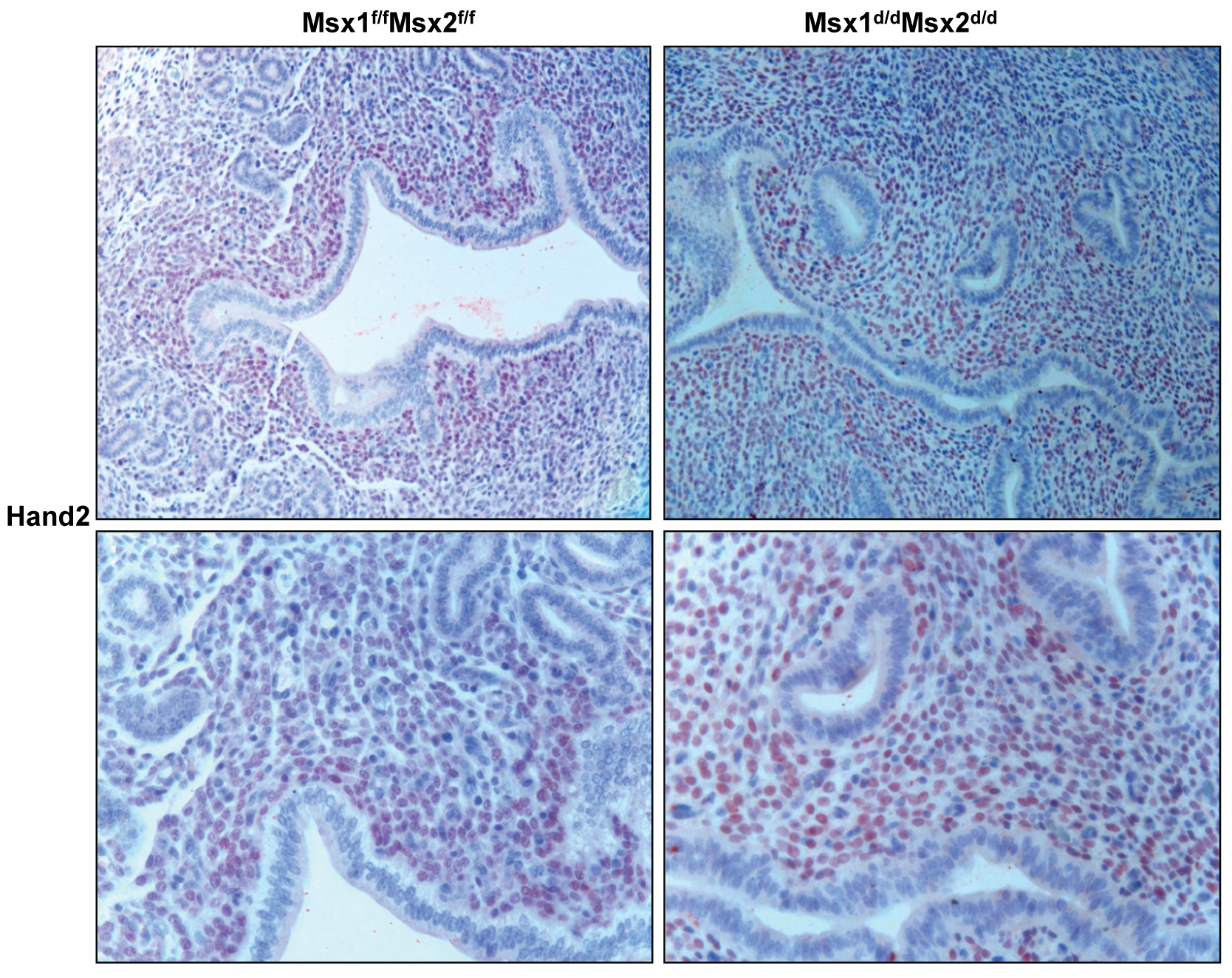

Supplement: Figure S6 — Hand2 expression in Msx1d/dMsx2d/d uteri. The level of Hand2 in uterine sections of Msx1f/fMsx2f/f (left panel) and Msx1d/dMsx2d/d (right panel) mice on day 4 of pregnancy was analyzed by IHC (Magnification: upper panel: 20×, lower panel: 40×). (TIF) [file pgen.1002500.s006.tif]
